# Ecological Approach to Understanding Superinfection Inhibition in Bacteriophage

**DOI:** 10.3390/v13071389

**Published:** 2021-07-17

**Authors:** Karin R. H. Biggs, Clayton L. Bailes, LuAnn Scott, Holly A. Wichman, Elissa J. Schwartz

**Affiliations:** 1 School of Biological Sciences, Washington State University, Pullman, WA 99164, USA; karin.biggs@wsu.edu (K.R.H.B.); clayton.bailes@wsu.edu (C.L.B.); 2 Department of Biological Sciences, University of Idaho, Moscow, ID 83844, USA; lscott@uidaho.edu (L.S.); hwichman@uidaho.edu (H.A.W.); 3 Institute for Modeling Collaboration and Innovation, University of Idaho, Moscow, ID 83844, USA; 4 Department of Mathematics & Statistics, Washington State University, P.O. Box 643113, Pullman, WA 99164, USA

**Keywords:** bacteriophage, superinfection, inhibition, competition, ΦX174 reduction effect, virus population dynamics

## Abstract

In microbial communities, viruses compete with each other for host cells to infect. As a consequence of competition for hosts, viruses evolve inhibitory mechanisms to suppress their competitors. One such mechanism is superinfection exclusion, in which a preexisting viral infection prevents a secondary infection. The bacteriophage ΦX174 exhibits a potential superinfection inhibition mechanism (in which secondary infections are either blocked or resisted) known as the reduction effect. In this auto-inhibitory phenomenon, a plasmid containing a fragment of the ΦX174 genome confers resistance to infection among cells that were once permissive to ΦX174. Taking advantage of this plasmid system, we examine the inhibitory properties of the ΦX174 reduction effect on a range of wild ΦX174-like phages. We then assess how closely the reduction effect in the plasmid system mimics natural superinfection inhibition by carrying out phage–phage competitions in continuous culture, and we evaluate whether the overall competitive advantage can be predicted by phage fitness or by a combination of fitness and reduction effect inhibition. Our results show that viral fitness often correctly predicts the winner. However, a phage’s reduction sequence also provides an advantage to the phage in some cases, modulating phage–phage competition and allowing for persistence where competitive exclusion was expected. These findings provide strong evidence for more complex dynamics than were previously thought, in which the reduction effect may inhibit fast-growing viruses, thereby helping to facilitate coexistence.

## 1. Introduction

Inhibitory mechanisms have a large impact on virus population dynamics [1,2]. The inhibition of bacteriophage infection can lead to subsequent viral evolution and escape from that inhibition. Mechanistically, virus-mediated inhibitory mechanisms evolve to reduce sister viruses’ capacity to infect, appropriately replicate, or efficiently release progeny. They may also evolve in order to enhance competition for control of host resources. The conditions in which these inhibitory mechanisms evolve are most commonly characterized by high viral density and a relative scarcity of available hosts, i.e., when multiplicity of infection (MOI) is high. Such conditions drive direct competition between identical or closely related viral strains at all viral life stages, especially during the processes of adsorption and replication [3]. These changes lead to a shift away from r-selected reproduction strategies (in which species have a high growth rate but low survivability), and a shift toward K-selected reproduction strategies (in which species have a low growth rate and high survivability) [4]. Additionally, these strong selective pressures can give rise to the dedication of viral resources to the development of superinfection exclusion mechanisms.

Superinfection exclusion, a phenomenon in which a preexisting viral infection prevents a secondary viral infection, requires the dedication of resources to defense that often comes at a fitness cost. However, in the presence of competition, the advantage of defense can be substantial. This auto-inhibitory phenomenon has evolved independently in a wide variety of prokaryotic and eukaryotic viruses [5,6,7,8,9,10,11]. Superinfection inhibition encompasses both superinfection exclusion and superinfection immunity (referring to a block or resistance to secondary infection after entry) collectively [12,13] and occurs due to a variety of mechanisms [14,15,16]. The bacteriophage ΦX174 exhibits an auto-inhibitory phenomenon known as the reduction effect, which is hypothesized to mimic a superinfection inhibition mechanism [17]. The exact mechanism of the reduction effect is not completely understood, but it is linked to underlying processes during virus replication, and so an overview of the ΦX174 infection cycle is essential for a more complete interpretation of the data presented here.

The lytic bacteriophage ΦX174 is perhaps the best-known virus in the *Microviridae* family and has been used widely as a model system for experimental evolution [18,19]. ΦX174 has a circular, single-stranded, positive-sense genome with a length of 5386 nucleotides that is contained within a tailless icosahedral capsid [20,21]. The infection cycle of ΦX174 begins with attachment to lipopolysaccharides on the host cell (*E. coli*) surface, followed by the injection of viral DNA, replication inside the cell, and cell lysis to release the phage progeny. In virus replication, the phage’s single-stranded genome is first converted into a double-stranded circular replicative form (called RF1) through the use of 13 host proteins [20,21]. Next, RF1 DNA is amplified, requiring a viral protein (gpA) and the host *rep* protein. Thirdly, single-stranded DNA (ssDNA) is synthesized and packaged into viral procapsids, necessitating viral proteins gpA and gpC. After the assembly of the capsid and insertion of the new viral genome, the new phages are released during cell lysis.

The viral pilot protein gpH is of particular interest, as it has several functions during the viral lifecycle [22,23,24,25]. Structurally, a molecule of gpH is incorporated as a monomer at each of the viral capsid’s 12 vertices. Following attachment, genome translocation is mediated by gpH. Ten to twelve gpH molecules oligomerize to form a DNA translocating tube that pilots the single-stranded genome across the host cell wall [22,23]. In addition, gpH is thought to play a role in the early stages of replication. It is hypothesized that gpH may enter the cell along with the phage genome and aid in the conversion of ssDNA to RF1 [26]. Finally, de novo gpH production has been shown to be required for efficient viral coat protein synthesis [27].

The ΦX174 reduction effect is an auto-inhibitory phenomenon specific to ΦX174 and its related phages. In this phenomenon, which was first described by van der Avoort et al. in 1982 [17], cells that were once permissive to ΦX174 become resistant to infection when a plasmid containing a fragment of the ΦX174 genome has been introduced into the cells. Van der Avoort et al. identified the specific segment of the ΦX174 genome responsible for the reduction effect as a 495-base sequence that spans the C-terminal end of pilot protein gene H, the N-terminal end of the replicative form (RF) replication gene A, and the 63-base H–A intergenic region. They hypothesized that this sequence causes this inhibition through DNA–protein binding that blocks a necessary site for the conversion of infecting genomes to RF1 DNA. Evidence for this hypothesis includes a smaller than expected quantity of RF1 DNA formed [17]. The inhibition might occur either through a direct interaction, such as by preventing the formation of RF1 DNA, or indirectly, by sequestering a necessary replication factor in the host cell.

Further work on the reduction effect has assessed the ability of ΦX174 to overcome inhibition by the reduction plasmid, indicating that recovery was possible via as little as a single point mutation in the phage genome within the H gene [28]. Additional studies established that the reciprocity of inhibition between reduction sequences in *Microviridae* phages is possible [29], but not required. For instance, the reduction sequence from bacteriophage G4 is capable of inhibiting the growth of both G4 and ΦX174, but the reduction sequence from ΦX174 is only capable of inhibiting the growth of ΦX174. The asymmetric reciprocity of inhibition shown between these species implies that the selective pressures that wild isolates face may vary dramatically.

Here, we expanded this work to examine the reduction effect in wild ΦX174-like phages and to better understand the role that the reduction effect and superinfection inhibition may play to confer a selective advantage in microbial competition. First, we quantified how inhibitory the ΦX174 reduction effect is in wild ΦX174-like phages. Next, we assessed the reciprocal inhibitory capacity of various members of the ΦX174-like clade by determining the levels of inhibition that the reduction sequences from four wild ΦX174-like phages exerted upon each other. Finally, we sought to investigate the possible role of the reduction effect on the persistence of multiple viral species in microbial communities. To accomplish this aim, we measured the population frequencies of pairs of ΦX174-like phages that were grown together over time in continuous culture, and compared them to expectations based upon each phage’s fitness and the inhibitory strength of its reduction sequence. Our results revealed that some pairs of phage–phage competitions could be predicted based upon low MOI fitness. In others, however, the interplay between low MOI fitness and the reduction effect is more likely to govern the outcome, leading to coexistence rather than exclusion. Thus, the reduction effect may serve to inhibit superinfection by an identical or closely related phage, conferring a competitive advantage that maintains the coexistence of species with lower growth rates. The broader implication of this work highlights the complex dynamics governing bacteriophage competition in their natural environments.

## 2. Materials and Methods

### 2.1. Isolates and Media

All isolates for the wild phage assays, including our laboratory strain of ΦX174, have been previously sequenced and described [30]. Annotated genomic sequences can be found in GenBank with the following accession numbers: ΦX174, AF176034.1; S13, AF274751.1; WA4, DQ079893.1; WA10, DQ079894.1; WA11, DQ079895.1; ID1, DQ079880.1; ID22, DQ079881.1; ID34, DQ079882.1; ID45, DQ079883.1; NC1, DQ079884.1; NC5, DQ079885.1; NC7, DQ079886.1; NC11, DQ079887.1; NC16, DQ079888.1; NC37, DQ079889.1; NC41, DQ079890.1; NC51, DQ079891.1.

We also engineered two phages, each with two missense mutations in the reduction sequence portion of the H gene, to create constructs of ΦX174 with the H reduction sequence of either WA11 (ΦXwa11) or NC16 (ΦXnc16). ΦXwa11 contained the H mutations I297F and I301V. ΦXnc16 contained the H mutations H275L and H299Y. Engineered phages were created by Golden Gate assembly of the phage genomes and transformation into host cells according to the methods described in Faber et al. [31].

For liquid fitness assays and chemostat assays, a modified Luria–Bertani medium called ΦLB was used. ΦLB consists of 5 g/L yeast extract, 10 g/L tryptone, and 10 g/L NaCl, supplemented with 2mM CaCl_2_. For solid plates, 15 g/L Bacto agar was added to ΦLB, and 7 g/L Bacto agar made the semisolid top agar.

### 2.2. Reduction Plasmids

The ΦX174 reduction effect plasmid described by van der Avoort et al. [17] contains the 495-base fragment spanning the end of gene H, the beginning of gene A, and the 64-base H–A intergenic region (bases 3705–4200). This fragment from ΦX174 was cloned into the same plasmid and at the same insertion site as described by van der Avoort et al.; the insertion was confirmed by sequencing. The plasmid containing the ΦX174 reduction sequence is referred to here as pΦX174rs. The control plasmid without the ΦX174 insert is pACYC177. Additional reduction sequence plasmids were constructed for wild phage isolates by aligning reduction sequences for WA4, WA11, NC16, and ID34 strains, and then following the same PCR, cloning, and sequencing protocol described above. These plasmids are referred to as pWA4rs, pWA11rs, pNC16rs, and pID34rs, respectively. Once in the cells, these cloned sequence inserts were transcribed using the Amp-R (β-lactamase) promoter present in pACYC177.

### 2.3. Plaque Assays

**Figure 1:**
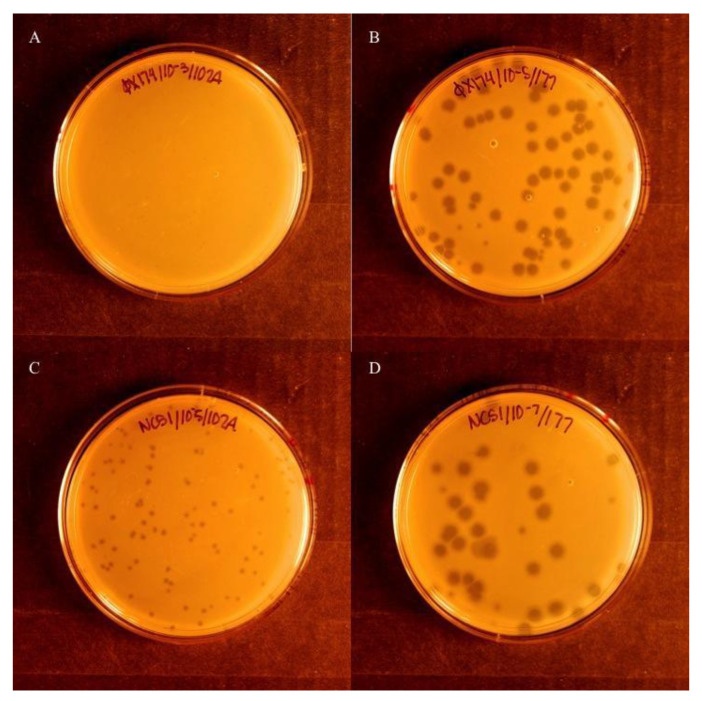
Visual comparison of phage isolates showing susceptibility to the reduction effect. (**A**) ΦX174 grown in the presence of the reduction effect plasmid, pΦX174rs. (**B**) ΦX174 grown in the presence of the control plasmid, pACYC177. (**C**) NC51 grown with pΦX174rs. (**D**) NC51 grown with pACYC177. ΦX174 grown in the presence of pΦX174rs (**A**) is susceptible to the reduction effect when compared to the permissive host with pACYC177 (**B**). NC51, in contrast to ΦX174, is not as susceptible, as evidenced by the visible plaques when grown on pΦX174rs (**C**). In NC51, plaques with pΦX174rs (**C**) are smaller than those seen on the permissive host with pACYC177 (**D**).

Blind plaque assays were conducted initially to identify susceptibility to the reduction effect. Wild phage samples were coded and plated in parallel in cells containing either the control plasmid pACYC177 or the reduction plasmid pΦX174rs, and incubated at 37 °C for 4 h, after which plates were scored for visible plaques. Five replicates were conducted, and samples were decoded. Samples that did not show plaques in any replicates were deemed susceptible to the reduction effect. If five of five replicates showed plaques, the phage strain was considered insusceptible to the reduction effect. If at least one of five replicates showed plaques, the strain was considered semi-susceptible (Figure 1).

To further quantify the level of inhibition of the reduction effect on different phages, liquid culture fitness assays were conducted. Results from the liquid assays indicated that the plaque assays were not always a good indication of the degree of inhibition. Due to this inconsistency, along with the binary nature of the plaque assay, this approach was abandoned in favor of liquid culture fitness assays.

### 2.4. Fitness Assays

Fitness assays were designed to measure phage fitness as the number of phage doublings per hour in a homogeneous liquid culture. This low-density assay for growth rate has been described previously [32]. High-density assays were found to be unreliable and inconsistent. Briefly, *E. coli* C cells containing the appropriate plasmid were grown in a 125 mL flask containing 10 mL ΦLB in a shaking water bath for 1 h at 37 °C to a concentration of ~2 × 10^8^ cfu/mL. Phages were then added so that the flask contained between 10^3^ and 10^4^ phage/mL. The initial number of phages was chosen to assure that the final density of phages was not allowed to exceed cell density and keep the multiplicity of infection (MOI) < 1. Phages were grown for 40 min with shaking. Final time samples (T_final_) were taken onto chloroform to stop bacterial growth. Samples from time points 0 and 40 min were titered, and doublings per hour were determined by the following formula:

$$fitness=\frac{log_{2}\left(T_{final}\right)-log_{2}\left(T_{initial}\right)}{time\;elapsed\;in\;hours}$$

Assays were conducted with both the control plasmid (pACYC177) and the ΦX174 reduction plasmid-containing (pΦX174rs) cells in triplicate unless otherwise noted. A number of phage types were assayed, including ΦX174, the related laboratory isolate S13, and 15 wild phage strains [30]. For the reciprocal fitness experiment, assays were conducted with the control plasmid (pACYC177) and each of the reduction plasmid-containing (pΦX174rs, pWA4rs, pWA11rs, pNC16rs, and pID34rs) cells in triplicate unless otherwise noted. The phage types assayed for fitness were WA4, WA11, NC16, ID34, and ΦX174 with each of the six plasmids listed above. For these assays, titers were assessed using *E. coli* C, a fully permissive bacterial strain. We measured the inhibitory capacity of each wild phage’s reduction sequence as the percent reduction in fitness between the permissive host (pACYC177) and the reduction sequence assayed:

$$\%\;inhibition=\frac{fitness\left(pACYC177\right)-fitness\left(rs\right)}{fitness\left(pACYC177\right)}*\;100.$$

### 2.5. Chemostat Competition Assays

To examine the competitive fitness of each phage in a continuous culture, a two-stage chemostat was used (Supplementary Figure S1). ΦLB media flowed through a peristaltic pump, set at a rate of 7–10 mL/h, into the first of two 100 × 15 mm glass test tubes via silicone tubing [32,33]. The first tube was inoculated with 500 µL of log phase *E. coli* C cells. Cells and media then flowed into the second test tube, which contained the phage population. From there, media, cells, and phage were pulled out using an aquarium pump into a waste container to maintain a continuous population volume [32,33]. Both the bacteria and phage tubes were submerged in a water bath held at 37 °C. The lids of both tubes contained a port to allow for the inoculation of each tube, and the second tube also contained a port for the collection of samples. Since the system allows for one-way flow of fresh media and the continuous introduction of new cells, bacterial evolution was minimized, as was bacteria–phage coevolution.

Competition assays were conducted for six separate pairings of phages: ΦX174 vs. WA11, ΦX174 vs. NC16, WA11 vs. NC16, ΦX174 vs. ΦXwa11, ΦX174 vs. ΦXnc16, and ΦXwa11 vs. ΦXnc16. Each set of chemostats was run in triplicate for 6–24 h each. The chemostat was initially inoculated with 500 µL of cells growing at log phase (~2 × 10^8^ cfu/mL) into the first tube. The chemostat was allowed to run for half an hour before phages were added to the second tube. Equal parts of both competing phages (~1 × 10^4^ of each phage) were then added to the second tube. Samples were taken from the chemostat at hours 2, 4, 6, and, in some cases, 24. Samples were filtered at 0.2 µm to remove cells. At the end of each chemostat run, samples from the unused media and the cell tube were each taken and plated on ΦLB agarose plates to verify that contamination had not occurred. Twenty-four-hour samples were not available for all competitions, and due to the likelihood of evolution occurring by 24 h [33,34], the hour 6 samples are used in the final analyses. It is worth noting that for the available 24-h samples, there were no major differences in the overall result compared to the 6-h samples.

### 2.6. Quantification of Competition Assay Phage Populations

The relative abundance of each competing phage at each time point was estimated using MiSeq Illumina sequencing (Illumina Inc., San Diego, CA, USA). The MiSeq reads were mapped to each of the bases differing between the competing phages. The percentage of each unique base was averaged at each time point to give an estimate of the relative proportion of each phage. For any given time point, there was less than a 5% difference between the estimates of the phage proportions.

The predicted percentage, *P*, of the dominant phage for each chemostat (gray dotted lines in Figures 7 and 8) was based on the growth data of each phage grown without the presence of the reduction sequence and was calculated as follows: *P_t+1_ = P_t_ × 2^x^*, where *x* is the control fitness, and *t* is time in hours. These predictions were based on the uninhibited growth rate only, and did not take competitive ability into account. The proportion of each competing phage in the population was then determined by calculating the percentage of each phage of the total.

### 2.7. Sequence Alignments

Sequences were aligned via the ClustalW method and divergences were calculated using the MegAlign program in the Lasergene suite (DNASTAR, Madison, WI, USA).

### 2.8. Sequencing

The sequences of the phage genomes and plasmid inserts were ascertained before use in fitness assays. Phage genomes were PCR amplified in overlapping halves and reactions were cleaned up on Qiaquick columns (Qiagen, Hilden, Germany). Sequences of the PCR products were visualized on an ABI capillary sequencer using BigDye terminator chemistry (Thermo Fisher, Waltham, MA, USA) and assembled with the SeqMan program of Lasergene. Ambiguities and gaps were re-sequenced, using alternative primers when needed. Plasmid insert sequences were obtained by terminator labeling using forward and reverse primers to the plasmids.

Phage populations from the chemostat competition assays were sequenced using MiSeq Illumina sequencing. To prepare the samples for sequencing, two rounds of PCR were run. The first round amplified an approximately 500-base segment of the C-terminal end of the H gene and added the adaptor sequence to each end necessary for the second round of PCR. The primers for the first round of PCR included 0–2 bases inserted as spacers between the adaptor and H portions. First round PCRs were column purified and products were checked by electrophoresis. The second round of PCR reactions involved further amplifying the selected genome fragment as well as attaching a unique Illumina adaptor to the ends of each sample. Samples were again cleaned and checked for concentration by electrophoresis. Illumina sequencing was performed in the IBEST Genomic Resources Core at the University of Idaho. All primers used for amplifying and sequencing phage DNA are given in Supplementary Table S1.

## 3. Results

### 3.1. Characterization of Growth in ΦX-Like Clade: Quantification of Fitness

To obtain a quantitative assessment of susceptibility to the reduction effect, liquid culture fitness assays were used to measure the phage growth rate (doublings per hour). Results from the fitness of phages in cells containing the control plasmid, pACYC177, were compared to those containing the reduction plasmid, pΦX174rs. A wide range of inhibition was observed (Table 1).

**Figure 2:**
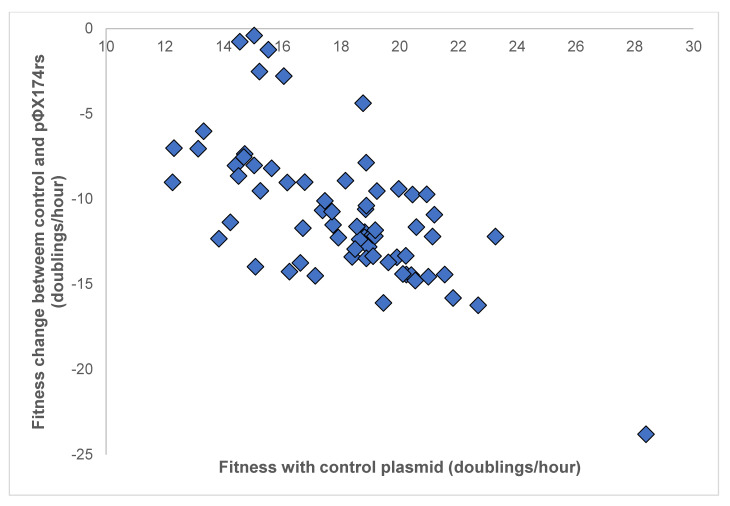
Fitness of phages grown in cells containing the control plasmid compared to the difference in fitness between growth in cells with the pΦX174rs plasmid and the control plasmid. Each point indicates the result of a single set of fitness assays. The weighted Spearman’s correlation analysis shows a moderate negative correlation (r = −0.65).

All bacteriophage strains exhibited reduction, i.e., a loss in fitness as measured in doublings/hour, when grown in the presence of the plasmid pΦX174rs. Assays of the strains grown in the presence of the reduction sequence plasmid showed an average fitness of 7.66 doublings/hour, compared to the average fitness of the strains with the control of 17.45 doublings/hour, representing a 56% average reduction in fitness. To visualize the fitness reduction as a function of a phage’s control fitness, Figure 2 shows the fitness of each phage with the control plasmid compared with the fitness change between growth with the control plasmid and the reduction sequence plasmid. A weighted Spearman’s correlation analysis (weighted by the number of samples of each phage strain) showed a moderate negative correlation (r = −0.65) between fitness change and control, suggesting that strains with higher control fitness indicate larger fitness reductions in the presence of the reduction plasmid.

**Figure 3:**
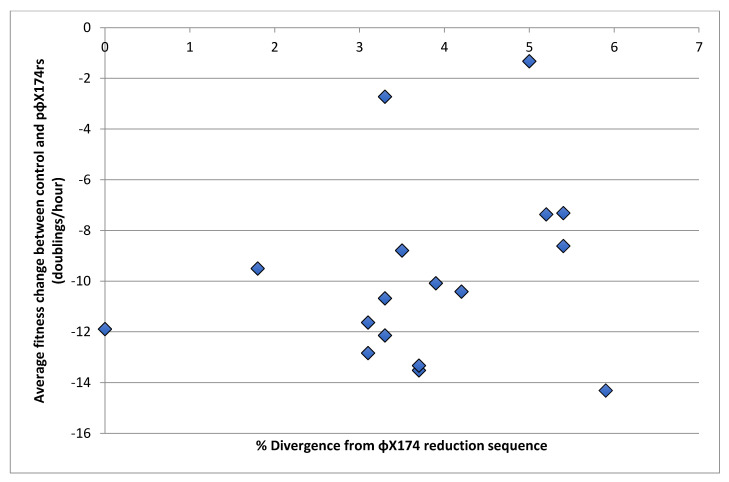
Difference between the fitness of wild phages with the control plasmid and the ΦX174rs plasmid plotted against the divergence of those phages from ΦX174 in the region of the reduction sequence. Spearman’s correlation analysis between percent divergence and fitness change indicated that overall divergence does not have an impact on the inhibition by the ΦX174 reduction sequence (r = 0.23).

To determine if there is a relationship between reduction sequence homology and fitness loss on the reduction sequence plasmid, sequence divergence of the wild phage strains from the ΦX174 reduction sequence was compared to fitness change using correlation analysis. Here, we show the average fitness change between control and the ΦX174 reduction sequence for each phage versus the percent divergence from the 495-base ΦX174 reduction sequence (Figure 3, Table 1). Divergence was found to be uncorrelated with fitness change, indicating no apparent association between a strain’s overall difference from the ΦX174 reduction sequence and its fitness under the ΦX174 reduction effect (r = 0.23, Spearman).

### 3.2. Reciprocity of Inhibition by the Reduction Sequences of Other ΦX-Like Phages

**Figure 4:**
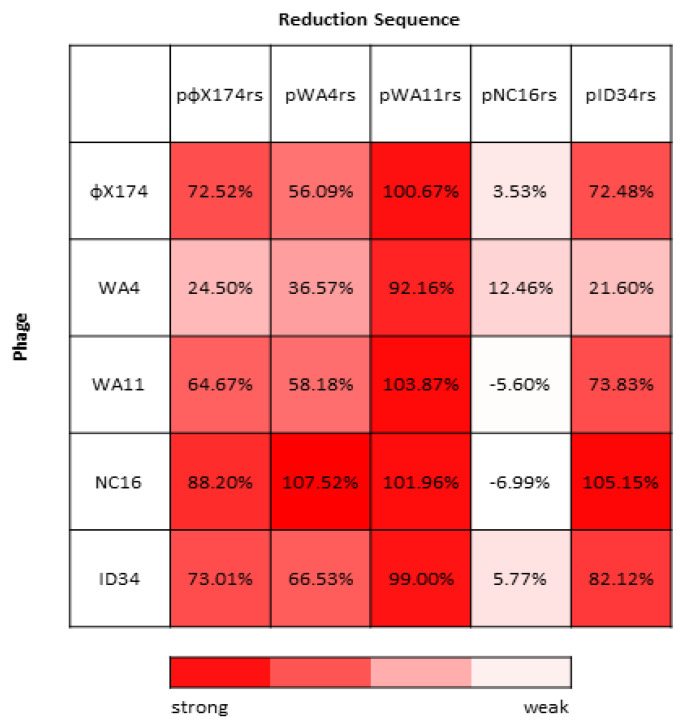
Percent inhibition (calculated as described in Methods) for all assayed phages grown in the presence of each reduction sequence. Least inhibited phage–reduction sequence pairings are shown in white, while the most inhibited phage–reduction sequence pairings are shown in red.

Work by van der Avoort et al. [29] found an asymmetric reciprocity in the pattern of inhibition between the ΦX174 and G4 reduction sequences, suggesting the possibility of a complex network of competitive interactions between these phages. We further characterized this phenomenon by constructing reduction effect plasmids containing the reduction sequence of four wild ΦX174-like phages and then measuring the fitness of ΦX174 and each of these four phages against each of their reduction plasmids. When the alternative reduction plasmids are utilized, we see that there is broad variability in inhibitory capacity among each wild phage’s reduction sequence (Figure 4).

The WA11 reduction sequence (pWA11rs), for example, almost completely inhibits all assayed phages (between 92.16% and 103.87% inhibition), while the NC16 reduction sequence (pNC16rs) inhibits virtually none (between −6.99% and 12.46% inhibition). The remaining reduction sequences (pΦX174rs, pWA4rs, and pID34rs) lie somewhere in between and largely depend on the infecting phage (Figure 4). The NC16 phage appears to be broadly inhibited, except when assayed with its own reduction sequence, pNC16rs. The WA4 phage appears to be broadly less inhibited, except when assayed with the pWA11rs plasmid.

### 3.3. Clues within the Sequence: Amino Acid Changes in H

**Figure 5:**
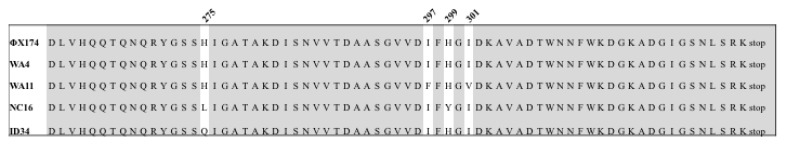
Alignment of amino acid sequences of the reduction sequence portion of ΦX174 and ΦX174-like wild phages protein H sites 259–329. Differences occur at amino acids 275, 297, 299, and 301 and are highlighted.

As no relationship between sequence divergence and fitness loss was observed, we further explored a hypothesis in which specific mutations allow adaptation to inhibition. To determine if the inhibitory power of a reduction sequence plasmid is related to its specific genetic sequence, all five wild phages’ reduction sequences were aligned and analyzed (Figure 5). We observed four amino acid sites in the plasmids that were indicators of the inhibitory capacity of a reduction sequence: protein H residues 275, 297, 299, and 301.

Two of these amino acid changes in protein H were unique to the strongly inhibitory plasmid pWA11rs: I297F and I301V. Being the only differences from ΦX174rs within this region, they are therefore associated with an increased inhibitory capacity. Another two protein H amino acid changes were unique to NC16, the particularly poor inhibitor: H275L and H299Y. Again, these two mutations are the only changes from the ΦX174rs within this region and are therefore associated with a diminished inhibitory capacity. Another H residue change, H275Q, is seen in our moderately inhibitory pID34rs plasmid and occurs at the same site as one of the pNC16rs mutations.

### 3.4. Competition of ΦX174-Like Phages in Continuous Culture

In order to link the inhibition findings presented above with the ecological context of phage–phage competition, competition assays in chemostats were conducted using these phages: WA11 (strong inhibitor), NC16 (weak inhibitor), and ΦX174 (moderate inhibitor). Additionally, two engineered phages were used. Each engineered phage was identical to ΦX174, but with two genomic missense mutations to match the reduction sequence of either WA11 (ΦXwa11) or NC16 (ΦXnc16) (Figure 5). The resultant phage ΦXwa11 contained the protein H mutations I297F and I301V. Phage ΦXnc16 contained the protein H mutations H275L and H299Y.

**Figure 6:**
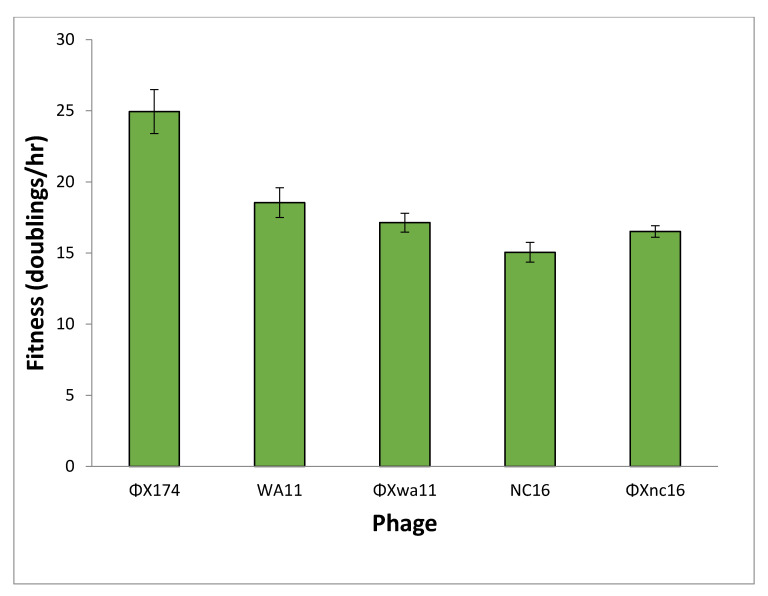
Fitness of each phage grown in *E. coli* C cells without plasmids. Both engineered phages are identical to ΦX174 except for two amino acid changes to match the reduction sequences of WA11 (ΦXwa11) and NC16 (ΦXnc16). Error bars indicate standard error.

The fitness values of the phages when grown alone were as follows: ΦX174, 25 doublings/h; WA11, 18.5 doublings/h; NC16, 15 doublings/h; ΦXwa11, 17 doublings/h; and ΦXnc16, 16.5 doublings/h (Figure 6). The two amino acid changes in each of the engineered phages resulted in a substantial drop in fitness compared to ΦX174. It is worth noting that these fitness values represent growth at MOI < 1 and thus represent the fitness possible when no competition for host cells is present.

Predictions for the outcome of each competition were based on two factors: low MOI fitness and the inhibitory properties of each phage’s reduction sequence (Table 2). Based on the fitness (Figure 6), it would be expected that ΦX174 would outcompete both the wild and engineered phages: ΦX174 has a fitness advantage of ~6.5–10 doublings per hour. If intrinsic fitness is the key driver in competition between phages, the expectation is that the majority population of each chemostat involving ΦX174 would be ΦX174, and that WA11 has a ~3.5 doublings/h advantage over NC16.

**Figure 7:**
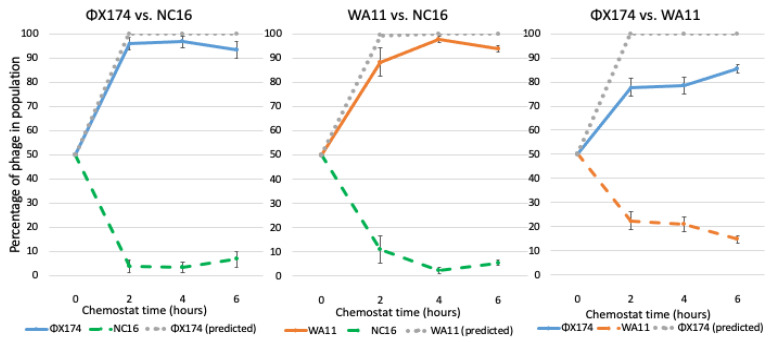
Population dynamics of ΦX174, WA11, and NC16 in pair competitions over time. All chemostats were inoculated at time zero with equal parts of each phage, as estimated by phage stock titers. Competitions were run in triplicate with samples taken at hours 2, 4, and 6. The gray dotted line indicates the predicted winning phage based on fitness. Error bars indicate standard error.

As expected based on both fitness and reduction sequence inhibition, when ΦX174 or WA11 were competed against NC16, the majority (>90%) of the resulting phages were either ΦX174 or WA11, respectively (Figure 7, Table 3). When ΦX174 was competed against WA11, however, ΦX174 was dominant, though WA11 made up 15% of the total population after 6 h, which was a larger portion of the total population than NC16 comprised when it was competed against ΦX174. Thus, the predictions based on fitness held true in all three of the phage–phage competitions. In the competition between ΦX174 and WA11, in which the predictions based on fitness or reduction sequence differed, however, a higher minority population was observed, suggesting an impact of the reduction sequence on the persistence of the minority population.

**Figure 8:**
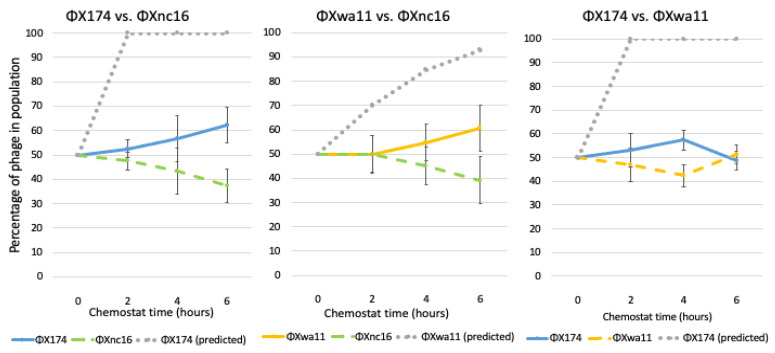
Population dynamics of ΦX174 and ΦX engineered phages in pair competitions over time. All chemostats were inoculated at time zero with equal parts of each phage, estimated by phage stock titers. ΦXwa11 is identical to ΦX174 except for two amino acid changes in protein H, I297F and I301V, to match the WA11 reduction sequence. ΦXnc16 is identical to ΦX174 except for two H amino acid changes, H275L and H299Y, to match the NC16 reduction sequence. Phage competitions were run in triplicate with samples taken at hours 2, 4, and 6. The gray dotted line indicates the predicted winning phage based on fitness. Error bars indicate standard error.

When ΦX174 and ΦXwa11 were each competed against ΦXnc16, a modest majority (>60%) of the resulting phages were either ΦX174 or ΦXwa11, respectively, as predicted based on both fitness and reduction sequence inhibition (Figure 8, Table 3). When ΦX174 was competed against ΦXwa11, however, each phage comprised approximately equal fractions of the population after 6 h. Thus, in the latter case, in which the predictions based on fitness and based on reduction sequence differed, more phage coexistence was observed. Given the large drop in control fitness of the engineered phages compared to ΦX174 (Figure 6), these findings suggest that the two amino acid differences each engineered phage possesses play a role in the phage–phage competition. This is especially true of ΦXnc16, which not only has the lowest control fitness of the competed phage, but contains the NC16 reduction sequence, which is only slightly inhibitory.

## 4. Discussion

The ΦX174 reduction sequence was first observed by van der Avoort et al. in 1982 [17]. The insertion of this fragment of the ΦX174 genome into a plasmid greatly reduced the fitness of ΦX174 in cells containing the plasmid. The reduction effect phenomenon was hypothesized to mimic superinfection exclusion, but it remains unclear whether this is an ecologically relevant phenomenon or simply a laboratory artifact. To evaluate the ecological relevance of the reduction effect, we used several approaches. We first examined the inhibitory properties of the reduction sequences of ΦX174 and various ΦX174-like wild phages to evaluate the degree and specificity of this inhibition. We then moved from the plasmid-based system to a two-phage competition-based continuous culture system to investigate the reduction effect in phage–phage competition more similar to that seen in nature.

### 4.1. Evaluating the Scope of the Reduction Effect: Wild Phages Panel

We first quantified the fitness of wild ΦX174-like phages grown alone and in the presence of the ΦX174 reduction effect, and in each case we saw inhibition with the ΦX174 reduction sequence plasmid (Table 1). A moderate negative correlation between control fitness and fitness change with the reduction plasmid was observed, suggesting that, in general, phages with higher control fitness experience greater fitness loss in the presence of the pΦX174rs plasmid (Figure 2). The phages assayed exhibited broad variability in how inhibited they were by the various reduction effect plasmids (Figure 4). However, several interesting patterns were observed. The pWA11rs plasmid is capable of inhibition that is much stronger than the other plasmids, almost completely inhibiting all of the phages assayed. WA4 is consistently less inhibited by the reduction effect plasmids than the other phage isolates. Conversely, the NC16 isolate is generally more inhibited by reduction effect plasmids than the other phages assayed. The NC16 reduction sequence (pNC16rs) is minimally inhibitory, and in the cases of WA11 and NC16, actually increased the fitness of the infecting phage.

### 4.2. Inhibitory Capacity Has a Genetic Basis

Sequence divergence from ΦX174 was next examined to determine if reduction sequence homology plays a role in fitness loss on the reduction sequence plasmid (Figure 3). No relationship was found between divergence within the reduction sequence region of gene H and fitness loss, suggesting that inhibition is not driven by the overall divergence within this region, but rather by differences in specific nucleotides. Two unique missense mutations in the H gene component of the WA11 reduction sequence, resulting in protein H changes I297F and I301V, are associated with the increased inhibition displayed by its reduction sequence. Two different missense mutations found in the NC16 reduction sequence, resulting in changes H275L and H299Y, are associated with a lack of inhibition (Figure 5). Interestingly, two of the wild isolates, NC16 and ID34, possess a mutation at residue 275 in protein H (H275L in NC16 and H275Q in ID34); we note that mutations at this location have been previously identified as recovery mutations from the reduction effect [28,35], though with different amino acid changes (H275D and H275R). Together, these findings suggest an underlying genetic determination of inhibition associated with the reduction effect.

### 4.3. Competition between Phages Is Influenced by Both Fitness and Inhibitory Ability

We then performed competition assays in continuous culture to assess the outcomes of competition between these phages. We chose the phages for pairwise competitions based upon the inhibitory properties of the wild phage reduction sequences along with each phage’s susceptibility to these reduction sequences. We identified certain trends (Figure 4): NC16 is a weak competitor, while WA11 is a strong competitor. Accordingly, we selected NC16, WA11, and ΦX174 for the competition assays. The results of these competition assays were compared to the predictions based on fitness assays and on the inhibition by the various reduction sequences (Table 2). Competition between ΦX174 and NC16 and between WA11 and NC16 represent our ‘controls’, as both the fitness and reduction sequence predictions give the same outcome. This is also the case for competition between ΦX174 and the engineered phage ΦXnc16, as well as for ΦXwa11 against ΦXnc16. The competitions between ΦX174 against WA11 and ΦX174 against ΦXwa11, however, represent our ‘test cases’, as the predicted fitness outcome and the predicted reduction sequence outcome differed in each case. The outcome of the competitions performed here met the expectation when both the fitness and reduction sequence predictions aligned. When these predictions differed, however, we found that the reduction sequence allowed for greater coexistence than was predicted based solely on the phage’s fitness (Table 3). This indicates the key role this sequence may play in phage–phage competition.

We expected ΦX174 as well as WA11 to dominate a competition with NC16, and this outcome was observed (Figure 7). With ΦX174 vs. WA11, however, predictions based on the fitness of these phages suggested that ΦX174 would reach 95% of the total population within an hour; while ΦX174 was the dominant phage by the end of the competition, the rate at which it overtook the population was far slower than predicted, suggesting WA11 possessed some means to counteract ΦX174’s higher fitness. We note that the WA11 reduction sequence, pWA11rs, was highly inhibitory to every phage tested, including itself (Figure 4). Coupled with relatively low fitness when compared to ΦX174, this suggests a tradeoff in competitive ability at the cost of overall fitness. In the case of ΦX174 vs. ΦXwa11, the changes in the reduction sequence resulted in both phages competing equally, despite a fitness difference of almost eight doublings per hour (Figure 8). Given that it was predicted that ΦX174 would eliminate ΦXwa11 well before six hours, and that the only differences between these two phages are the two amino acid changes in the reduction sequence, these data provide strong evidence that the reduction effect helps maintain the persistence of the less fit phage.

Similarly, NC16 was nearly eliminated when competed against WA11, despite WA11 having only a slight fitness advantage, which is consistent with the strong inhibitory capacity of pWA11rs (Figure 7). Yet, in the case of ΦXwa11 vs. ΦXnc16, despite ΦXnc16 having both a lower fitness and a less inhibitory reduction sequence, ΦXnc16 was unexpectedly able to persist throughout the experiment (Figure 8).

### 4.4. Ecological Implications: High Density Interaction and Superinfection Inhibition

When the reduction effect plasmids are used in a lab-based system to mimic the primary infection of permissive cells, any productive phage infections are functionally secondary infections. Thus, we here interpret the fitness results as the response of a secondary infector to the presence of another genome at the time of infection. In this way, our lab-based system mimics the superinfection that is a product of high-density interaction.

Indeed, previous work with the reduction sequence has hypothesized that the ecological function of the reduction effect is that of superinfection exclusion [17,29]. Under the hypothesis of superinfection exclusion, we would expect these presumptive secondary infectors to be completely inhibited by the primary infection. For most of the reduction sequence plasmids used here, we observed inhibition (i.e., superinfection inhibition), in which the presumptive secondary infectors produced fewer progeny phages per unit time, and thus exhibited lower fitness. Our results are consistent with the notion that the reduction effect phenomenon emerged as an evolutionary response to high-density interaction. It should also be noted that the wild phages utilized here were originally isolated from sewage treatment plants [30], where phages are ubiquitous. In this environment, competition and superinfection are common, leading to potentially many unique ecological and evolutionary histories among phage strains. Given this scenario, the variation in inhibitory capacity observed in our study is not unexpected. It is worth noting that superinfection exclusion is not necessarily selected in phage populations for the sake of its ability to antagonize phage competitors. Instead, the underlying genes can be bases of pleiotropies and the ability to inhibit competitors is simply a beneficial by-product. This phenomenon has been seen in the phage T4 system in which the genes that allow for the lengthening of the latent period (to produce a larger burst size) when phage densities are low can enhance superinfection exclusion when densities are high [36,37].

In summary, the outcomes of the competitions shown here reflect both the phages’ fitnesses without competition and their inhibitory capacities. Cases where the two predictions based on low MOI fitness and reduction sequence inhibition diverged tended to result in an outcome somewhere in the middle. Although we clearly demonstrated that fitness at low MOI does not necessarily predict phage performance at high MOI, more research is needed to understand the complex interactions that occur during high MOI competition. For example, an additional factor that may influence competition is the formation of chimeric phages. If two phages are replicating within the same host cell, it is possible that structural proteins from the different phages may incorporate into either phage during capsid assembly, resulting in phages whose genomes do not fully indicate their resulting protein structure. While these other proteins would not be passed along during the next infection cycle, the inclusion of alternate proteins potentially plays a role during capsid attachment, the insertion of the phage genome, or the start of DNA replication. As a result, a less fit phage may persist in the high MOI environment of the chemostat. While the current study does not evaluate these specific implications, it is worth noting the potential effects that co-infection may have on persistence despite poor estimated competitive ability.

## 5. Conclusions

Overall, these findings provide an ecological context for the laboratory phenomenon of the reduction effect, and they provide strong evidence that the reduction effect modulates phage–phage competition. Further, these results support the hypothesis that the phenomenon proposed by van der Avoort et al. [17] is a mechanism of superinfection inhibition. Further studies in the future to experimentally assess bacteriophage population dynamics in general, and superinfection in particular, will build upon and advance the new results presented in this work.

## Figures and Tables

**Table 1:** Summary of fitness data for ΦX174-like clade.

Phage	Control Fitness ^1^ (Doublings/Hour)	SE	N	Fitness with Reduction Effect ^2^ (Doublings/Hour)	SE	N	Fitness Change with Reduction Effect Compared to Control ^3^ (Doublings/Hour)	Divergence from ΦX174 Reduction Sequence (%)
ΦX174	18.34	0.63	28	6.45	0.26	19	−11.89	0
S13	19.02	0.09	3	7.39	0.42	3	−11.63	3.1
WA4	15.21	0.44	3	13.89	0.40	3	−1.32	5
WA10	16.50	1.13	3	13.78	0.54	3	−2.72	3.3
WA11	18.54	1.05	3	6.40	0.68	3	−12.14	3.3
ID1	18.65	1.30	3	8.57	0.76	3	−10.08	3.9
ID22	19.43	1.21	3	6.59	0.43	3	−12.84	3.1
ID34	20.61	0.74	4	6.3	0.38	4	−14.31	5.9
ID45	18.9	1.12	3	8.49	0.58	3	−10.41	4.2
NC1	16.62	1.91	3	8.01	1.35	3	−8.61	5.4
NC5	15.13	0.33	3	6.34	0.55	3	−8.79	3.5
NC7	13.9	0.80	3	6.59	0.66	3	−7.31	5.4
NC11	12.9	0.33	3	5.54	1.21	3	−7.36	5.2
NC16	15.05	0.69	3	1.53	0.25	3	−13.52	3.7
NC37	16.81	0.16	3	3.48	0.79	3	−10.68	3.7
NC41	20.69	1.32	3	10.01	0.79	3	−10.68	3.3
NC51	20.31	0.73	3	10.81	0.28	3	−9.50	1.8

^1^ Average fitness of phages in the presence of the control plasmid pACYC177. ^2^ Average fitness of phages in the presence of the reduction effect plasmid pΦX174rs. ^3^ Change in fitness from control due to the presence of the reduction effect plasmid, calculated by subtracting the average control fitness from the average reduction effect fitness.

**Table 2:** Predicted outcomes of phage–phage competition.

Competition	Prediction Based on Phage Fitness ^1^	Prediction Based on Reduction Sequence
ΦX174 vs. NC16	ΦX174 95% of population after 1 h	ΦX174 is the better competitor
WA11 vs. NC16	WA11 95% of population after 2 h	WA11 is the better competitor
ΦX174 vs. WA11	ΦX174 95% of population after 1 h	WA11 is the better competitor
ΦX174 vs. ΦXnc16	ΦX174 95% of population after 1 h	ΦX174 is the better competitor
ΦXwa11 vs. ΦXnc16	ΦXwa11 93% of population after 6 h	ΦXwa11 is the better competitor
ΦX174 vs. ΦXwa11	ΦX174 95% of population after 1 h	ΦXwa11 is the better competitor

^1^ Estimated based on low MOI fitness assays, as described in Methods.

**Table 3:** Predicted and observed outcomes of phage–phage competition.

Competition	Prediction Based on Phage Fitness ^1^	Prediction Based on Reduction Sequence	Observed Results ^2^
ΦX174 vs. NC16	ΦX174 95% of population after 1 h	ΦX174 is the better competitor	ΦX174: 93% NC16: 7%
WA11 vs. NC16	WA11 95% of population after 2 h	WA11 is the better competitor	WA11: 94% NC16: 6%
ΦX174 vs. WA11	ΦX174 95% of population after 1 h	WA11 is the better competitor	ΦX174: 85% WA11: 15%
ΦX174 vs. ΦXnc16	ΦX174 95% of population after 1 h	ΦX174 is the better competitor	ΦX174: 62% ΦXnc16: 38%
ΦXwa11 vs. ΦXnc16	ΦXwa11 93% of population after 6 h	ΦXwa11 is the better competitor	ΦXwa11: 61% ΦXnc16: 39%
ΦX174 vs. ΦXwa11	ΦX174 95% of population after 1 h	ΦXwa11 is the better competitor	ΦX174: 49% ΦXwa11: 51%

^1^ Estimated based on low MOI fitness, as described in Methods. ^2^ Mean percentage of each phage in the 6-h population samples.

## Data Availability

Data is contained within the article or supplementary material.

## Supplementary Materials

The following are available online at https://www.mdpi.com/article/10.3390/v13071389/s1, Figure S1. Schematic depiction of the two-stage chemostat, Table S1. Primers used for amplifying and sequencing phage DNA.
