# Supplementary figures and images for: Ecological Approach to Understanding Superinfection Inhibition in Bacteriophage
Source: Viruses. 2021 Jul 17;13(7):1389. doi: 10.3390/v13071389 (PMC8310164; doi:10.3390/v13071389)

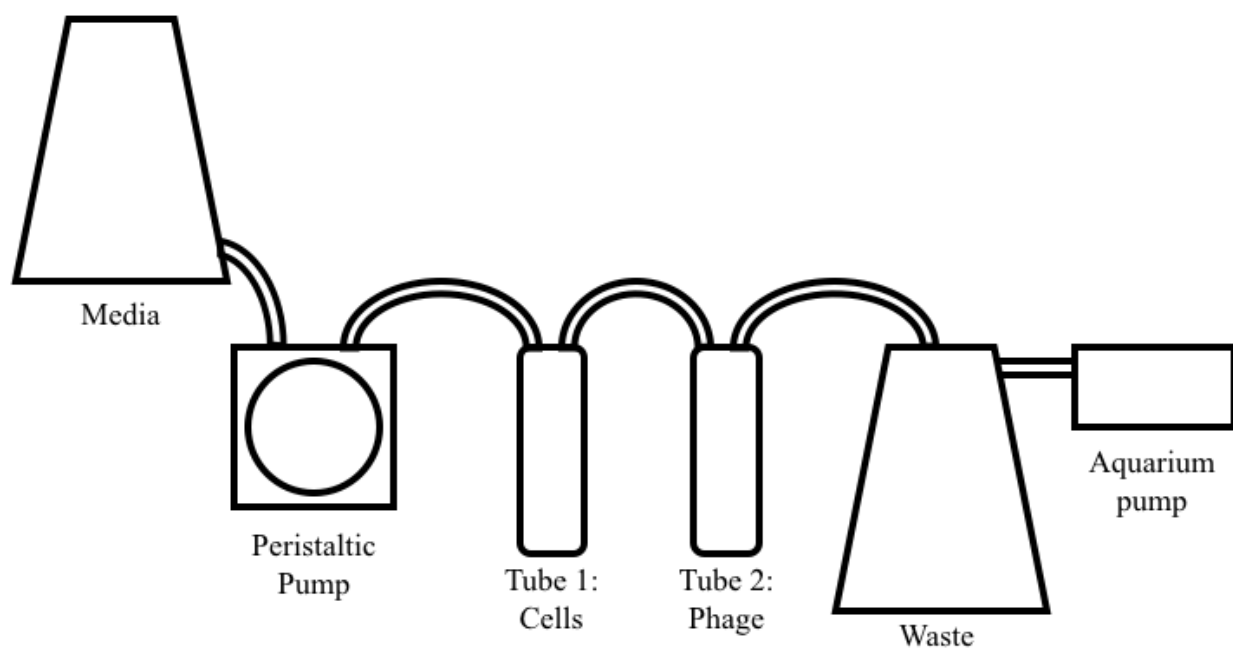

**Figure S1.** Schematic depiction of the two-stage chemostat.

Supplement: Supplementary file 1 [file viruses-13-01389-s001.zip › Supplementary Figure S1.pdf]
